# Sit-to-Stand Video Analysis–Based App for Diagnosing Sarcopenia and Its Relationship With Health-Related Risk Factors and Frailty in Community-Dwelling Older Adults: Diagnostic Accuracy Study

**DOI:** 10.2196/47873

**Published:** 2023-12-08

**Authors:** Juan D Ruiz-Cárdenas, Alessio Montemurro, María del Mar Martínez-García, Juan J Rodríguez-Juan

**Affiliations:** 1 Physiotherapy Department Faculty of Physiotherapy, Podiatry and Occupational Therapy Universidad Católica de Murcia Murcia Spain; 2 Cystic Fibrosis Association of Murcia Murcia Spain; 3 Physiotherapy Department Facultad de Medicina Universidad de Murcia Murcia Spain

**Keywords:** sarcopenia, power, calf circumference, diagnosis, screening, affordable, community dwelling, older adults, smartphone

## Abstract

**Background:**

Probable sarcopenia is determined by a reduction in muscle strength assessed with the handgrip strength test or 5 times sit-to-stand test, and it is confirmed with a reduction in muscle quantity determined by dual-energy X-ray absorptiometry or bioelectrical impedance analysis. However, these parameters are not implemented in clinical practice mainly due to a lack of equipment and time constraints. Nowadays, the technical innovations incorporated in most smartphone devices, such as high-speed video cameras, provide the opportunity to develop specific smartphone apps for measuring kinematic parameters related with sarcopenia during a simple sit-to-stand transition.

**Objective:**

We aimed to create and validate a sit-to-stand video analysis–based app for diagnosing sarcopenia in community-dwelling older adults and to analyze its construct validity with health-related risk factors and frailty.

**Methods:**

A total of 686 community-dwelling older adults (median age: 72 years; 59.2% [406/686] female) were recruited from elderly social centers. The index test was a sit-to-stand video analysis–based app using muscle power and calf circumference as proxies of muscle strength and muscle quantity, respectively. The reference standard was obtained by different combinations of muscle strength (handgrip strength or 5 times sit-to-stand test result) and muscle quantity (appendicular skeletal mass or skeletal muscle index) as recommended by the European Working Group on Sarcopenia in Older People-2 (EWGSOP2). Sensitivity, specificity, positive and negative predictive values, and area under the curve (AUC) of the receiver operating characteristic curve were calculated to determine the diagnostic accuracy of the app. Construct validity was evaluated using logistic regression to identify the risks associated with health-related outcomes and frailty (Fried phenotype) among those individuals who were classified as having sarcopenia by the index test.

**Results:**

Sarcopenia prevalence varied from 2% to 11% according to the different combinations proposed by the EWGSOP2 guideline. Sensitivity, specificity, and AUC were 70%-83.3%, 77%-94.9%, and 80.5%-87.1%, respectively, depending on the diagnostic criteria used. Likewise, positive and negative predictive values were 10.6%-43.6% and 92.2%-99.4%, respectively. These results proved that the app was reliable to rule out the disease. Moreover, those individuals who were diagnosed with sarcopenia according to the index test showed more odds of having health-related adverse outcomes and frailty compared to their respective counterparts, regardless of the definition proposed by the EWGSOP2.

**Conclusions:**

The app showed good diagnostic performance for detecting sarcopenia in well-functioning Spanish community-dwelling older adults. Individuals with sarcopenia diagnosed by the app showed more odds of having health-related risk factors and frailty compared to their respective counterparts. These results highlight the potential use of this app in clinical settings.

**Trial Registration:**

ClinicalTrials.gov NCT05148351; https://clinicaltrials.gov/study/NCT05148351

**International Registered Report Identifier (IRRID):**

RR2-10.3390/s22166010

## Introduction

Sarcopenia is an age-related muscle disease defined as the progressive loss of both muscle strength and muscle mass, which can lead to adverse health-related consequences [1]. Sarcopenia has been shown to be associated with clinical, lifestyle, and social risk factors such as depression, comorbidities, polypharmacy, smoking habit, low physical activity, low education level, and low socioeconomic status [2-6]. People with sarcopenia have an increased risk of developing frailty, falls, hospitalization, and mortality [7-10].

The prevalence of sarcopenia varies widely depending on the definition used, ranging from approximately 10% to 40% in community-dwelling older adults [11]. In order to allow the systematic and consistent identification of people with sarcopenia, the European Working Group on Sarcopenia in Older People-2 (EWGSOP2) published a consensus with specific cutoff points to facilitate sarcopenia diagnosis in research and clinical practice [1]. Accordingly, sarcopenia is determined by a reduction in both muscle strength and muscle quantity assessed with the handgrip strength test or 5 times sit-to-stand (5STS) test and by dual-energy X-ray absorptiometry or bioelectrical impedance analysis, respectively. However, in most settings, dynamometers are not widely available to measure muscle strength and access to muscle mass measurement techniques is limited or restricted [12]. In fact, several surveys have highlighted that most health care professionals do not diagnose sarcopenia mainly due to a lack of equipment and time constraints [13-15].

Given the growing importance of the early detection of sarcopenia to prevent subsequent adverse health-related consequences, a clinical, affordable, easy-to-use tool for sarcopenia screening and diagnosis might have implications for prognosis in community-dwelling older adults. In this context, several studies have reported that variables derived from kinematic analysis during a simple sit-to-stand transition could have the potential to discriminate between apparently healthy older adults and those with a history of falls, sarcopenia, or even frailty [16-19]. However, kinematic analysis during this functional task is characterized to be time-consuming and to require technical expertise and specialized equipment, such as 3D motion capture cameras, force plates, and inertial measurement units [17,18,20], which are usually restricted to laboratory settings. These issues represent barriers for health care professionals who require low-cost easy-to-use tools with automatic data processing and without the need for complex instrumentation.

Nowadays, the technical innovations incorporated in most smartphone devices, such as high-speed video cameras, provide the opportunity to develop specific smartphone apps [20,21] or web-based applications [22] for measuring kinematic parameters related with sarcopenia during sit-to-stand transition. This study was planned to develop and validate a video analysis–based app for detecting sarcopenia in community-dwelling older adults, using a muscle power kinematic parameter obtained during a simple sit-to-stand transition as a proxy of muscle strength and a calf circumference anthropometric parameter obtained with a simple measuring tape as a proxy of muscle quantity, according to the definition proposed by the EWGSOP2 guideline. Muscle power was selected as a proxy of muscle strength owing to its rapid decline during aging [23,24] and because it has been shown to be associated with several measures of physical function and sarcopenia determinants such as handgrip strength and the 5STS test in community-dwelling older adults with and without sarcopenia [21,25]. Additionally, calf circumference was selected as a proxy of muscle quantity owing to its association with appendicular skeletal mass (ASM) [26] and because it has been shown to improve the diagnostic accuracy of sarcopenia [27].

Since more than 5.4 billion people worldwide use mobile phones [28], the creation of a valid, affordable, and easy-to-use app that incorporates these 2 simple measures to detect sarcopenia could provide an excellent opportunity for health care professionals to assess sarcopenia status in community-dwelling older adults, where muscle strength dynamometers or muscle mass measurement techniques are usually limited or restricted [12].

Considering that sarcopenia diagnostic criteria should be based on their predictive value for hard outcomes [1], the aims of this study were to (1) create and validate a sit-to-stand video analysis–based app for diagnosing sarcopenia in community-dwelling older adults and (2) analyze its construct validity with clinical, lifestyle, and social risk factors such as depression, comorbidities, polypharmacy, smoking habit, low education level, low socioeconomic status, self-perceived health, falls, hospitalization, and frailty.

## Methods

### Study Design

A diagnostic accuracy study was carried out between February and October 2022 in 11 elderly social centers of the Region of Murcia (Spain). This study has been registered at ClinicalTrial.gov (NCT05148351).

### Ethical Considerations

The study complied with the Declaration of Helsinki and was approved by the Ethical Committee of the Catholic University of Murcia (CE022108).

### Eligibility Criteria

Community-dwelling adults aged 60 years or older were contacted via telephone or face-to-face to provide information to participate in the study. We excluded participants at risk of dementia (Mini-Cog score <3 points); those self-reporting cardiovascular problems (automatic defibrillator, pacemaker implantation, heart valve disease, and uncontrolled heart rhythm problems); those who were unable to stand up from a chair without assistance; and those with any health conditions that may affect the performance of functional tests, such as stroke sequelae, neuropathy, low back pain, and osteoarthritis.

### Procedures

After obtaining written informed consent, participants performed the following tests on the same day: (1) a single sit-to-stand test plus calf circumference measurement, (2) sarcopenia assessment, (3) frailty phenotype assessment, and (4) clinical interview.

The single sit-to-stand test plus calf circumference measurement was used as an index test to diagnose sarcopenia through a video analysis–based app. Sarcopenia assessment according to the EWGSOP2 guideline was used as the reference standard. Additionally, the Fried frailty phenotype and a clinical profile consisting of health-related risk factors assessed through a face-to-face interview were used for construct validity of the index test.

### Index Test: Sit to Stand App

A video analysis–based app (*Sit to Stand* app) installed on an iPhone 13 device running iOS 15.3 (Apple Inc) was created to analyze the sit-to-stand movement via a high-speed video recorded at 240 frames per second. The app was developed using Xcode and the Swift programming language (Apple Inc) for MacOS. The AVFoundation and AVKit frameworks (Apple Inc) were used for capturing, importing, and manipulating the high-speed videos. Then, the app was replicated and validated for running on Android in order to provide this tool for the 2 most widely used mobile operating systems [29]. The Android app was developed using Android Studio Chipmunk 2021.2.1 Patch 1, the Kotlin 1.5.21 programming language, and the Compose 1.0.1 UI framework for MacOS. For capturing, importing, and manipulating the high-speed videos, the CameraX 1.1.0 framework was used.

Prior to the execution of the single sit-to-stand test, the femur length (cm) and calf circumference (cm) of the participant were measured using an inelastic but flexible measuring tape. Femur length was measured as the distance between the superior aspect of the greater trochanter and femoral lateral condyle, while calf circumference was measured at the point of the greatest circumference on the nondominant leg in the sitting position with the knee and ankle joints at 90 degrees. A visual marker (colored sticker) was placed on the greater trochanter in order to identify the beginning and the end of the rising phase. To execute the test, participants sat on an adjustable height chair without footwear, with the hip, knee, and ankle joints at 90 degrees. Participants were instructed to stand from the chair “as fast as possible” with their arms crossed over their chest while the test was filmed simultaneously with the smartphone. The smartphone was placed horizontally on a 0.7-m-high tripod placed 3 m from the right or left side of the participant.

The variables provided from the app were rising time, vertical velocity, and vertical power. Rising time (s) was calculated from 2 selected frames by the user after analyzing the video. Vertical velocity (m/s) was automatically calculated from rising time and vertical distance. Vertical distance was introduced by the user into the app as femur length (cm), that is, the distance traveled from the sitting position at 90 degrees of the knee joint to full extension of the hip and knee joints. Finally, vertical power relative to body weight (W/kg) was estimated from the following regression equation (R^2^ adjusted=0.917; *P*=.04; standard error of estimate=0.45):


Power (W/kg) = 2.773 – 6.228 × *t* + 18.224 × *d*

where *t* is the rising time and *d* is the femur length. The *Sit to Stand* app has been previously validated against a 3D motion capture camera and force plates in community-dwelling older adults [20,21].

#### Data Analysis From the Sit to Stand App

The sit-to-stand movement can be divided into 3 phases: preparation phase, rising phase, and stabilization phase. The preparation phase starts when the trunk of the participant begins to accelerate forward and ends when the participant achieves seat-off. Then, the rising phase begins and ends when full extension of the knee and hip joints is achieved. In this phase, vertical velocity and power can be calculated. Finally, there is a stabilization phase where the hip extension velocity reaches zero and the stance of the body is achieved with quasi-static balance [30-32].

**Figure 1:**
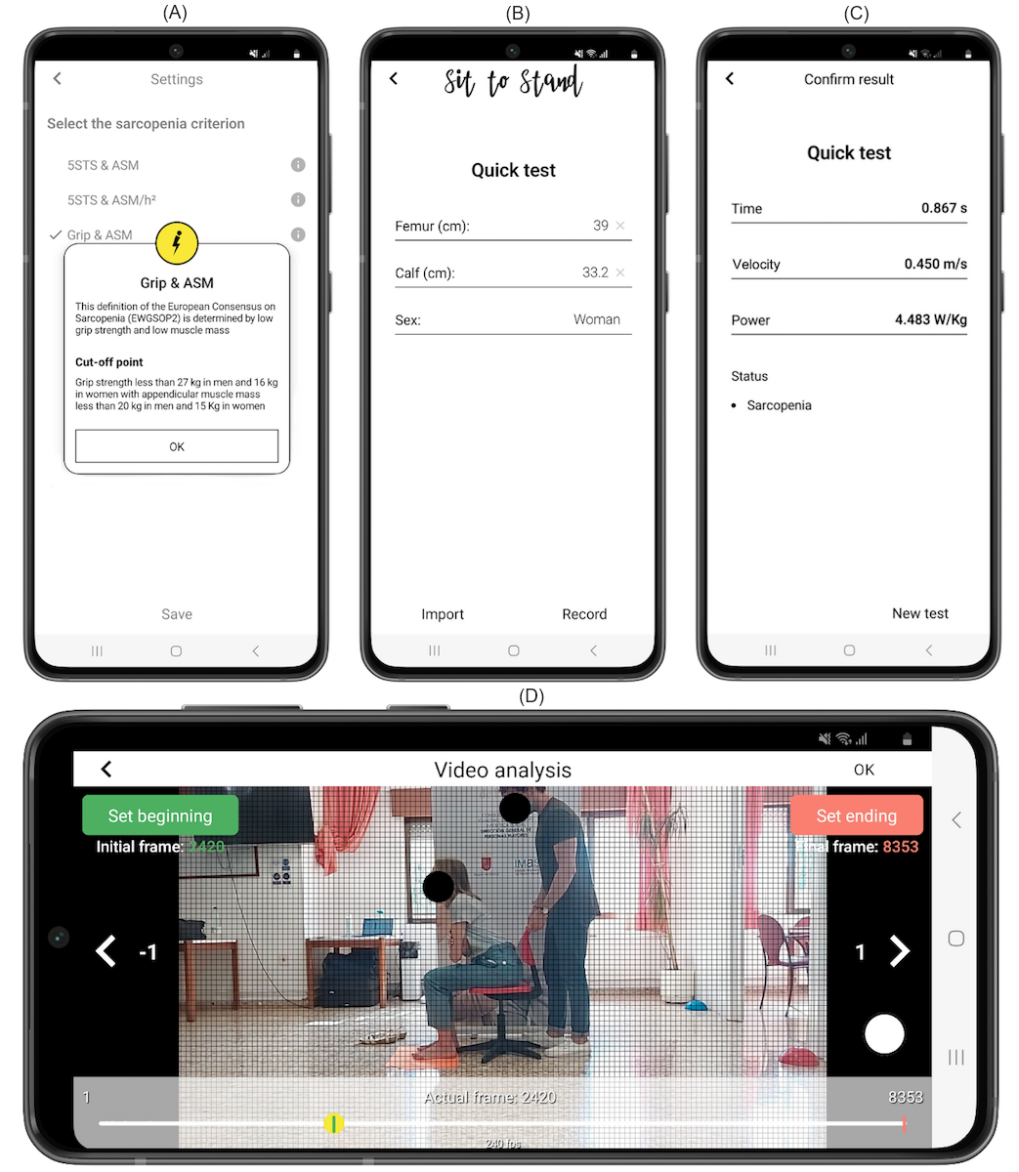
User interface of the Sit to Stand app running on the Android operating system. (A) Settings for selecting the different criteria proposed by the European Working Group on Sarcopenia in Older People-2 (EWGSOP2) guideline. (B) Participant characteristics. (C) Results from the app after video analysis. (D) Video analysis at 240 frames per second selecting the first frame corresponding to the beginning of the rising phase. Example of a woman with sarcopenia having a femur length of 0.39 m and calf circumference of 0.332 m.

To accurately determine the onset and the end of the rising phase, a visual grid for reference was built into the app as an overlay (Figure 1). Video analysis involved a manual process consisting of selecting 2 frames after video recording. The user selected the first frame and the final frame by pressing start and stop buttons, respectively. The onset of the rising phase was determined when the pelvis began to move forward after anterior trunk tilt, which was time-matched when the sticker was diagonally moved to the following square on the screen of the app. The end of this phase was defined when full extension of the hip and knee was achieved, which was time-matched when the sticker achieved the highest vertical point. A detailed explanation about video analysis can be found elsewhere [33]. Video analysis from the app was undertaken by a researcher blinded to sarcopenia assessment.

### Reference Standard: Sarcopenia by the EWGSOP2 Guideline

Sarcopenia was defined according to the recommendations proposed by the EWGSOP2 guideline [1]. Thus, a sarcopenia diagnosis was confirmed by the presence of both low muscle strength and muscle quantity. Low muscle strength was determined by using either handgrip strength or the 5STS test result as recommended by the EWGSOP2. Handgrip strength was evaluated using a handgrip digital dynamometer (Takei 5401, Takei Scientific Instruments Co, Ltd). Two repetitions from each hand were performed in the sitting position with the elbow flexed at 90 degrees, wrist in a neutral position, and thumb facing upwards. The maximal score was recorded, and a cutoff point for low muscle strength of <27 kg in men and <16 kg in women was used. For the 5STS test, participants were instructed to fully rise from a chair 5 times as fast as possible with their arms crossed over their chest. The time taken to sit down on the chair at the fifth repetition was recorded with a stopwatch. The cutoff point for low muscle strength was >15 s. Muscle quantity was assessed with regard to ASM (kg) or skeletal muscle index (kg/m^2^) through bioelectrical impedance analysis (TANITA MC-580, Tanita Corp). For muscle quantity calculation, the resistance index and reactance raw values were introduced in the validated equation by Sergi et al [34]. The cutoff points for low muscle quantity were <20 kg or <7 kg/m^2^ for men and <15 kg or <5.5 kg/m^2^ for women. Following the EWGSOP2 recommendations, 4 sarcopenia diagnostic criteria can be obtained (Table 1).

**Table 1:** Sarcopenia diagnostic criteria according to the European Working Group on Sarcopenia in Older People-2 (EWGSOP2) guideline.

Diagnostic criteria^a^	Low muscle strength	Low muscle quantity
SARC_HG+ASM_	Handgrip strength	Appendicular skeletal mass
SARC_HG+SMI_	Handgrip strength	Skeletal muscle index
SARC_5STS+ASM_	Five times sit-to-stand test	Appendicular skeletal mass
SARC_5STS+SMI_	Five times sit-to-stand test	Skeletal muscle index

^a^Cutoff points: handgrip strength (men <27 kg; women <16 kg); appendicular skeletal mass (men <20 kg; women <15 kg); skeletal muscle index (men <7 kg/m^2^; women <5.5 kg/m^2^); 5 times sit-to-stand test (>15 seconds for men and women).

### Construct Validity

#### Health-Related Risk Factors

Participants were systematically asked about the presence of depressive symptoms using the Spanish validated version of the Geriatric Depression Scale 5-items (cutoff point: ≥2), socioeconomic status (cutoff point: net salary <10,000 €/year [<11,000 US$/year]), education level (cutoff point: primary or less), presence of comorbidity (two or more chronic conditions), polypharmacy (five or more drugs/day), self-perceived health (cutoff point: very bad, bad, or fair), current smoking habit, presence of two or more falls in the last year, and hospitalizations in the last year. Additional information about the registered health-related outcomes can be found in the study protocol [35].

#### Frailty

Frailty syndrome was defined according to the Fried frailty phenotype assessment [36], which includes slowness (by usual pace 4-meter walking speed), exhaustion (by the Center for Epidemiologic Studies depression scale), unintentional weight loss (≥4.5 kg or ≥5% in the last year), weakness (by handgrip strength), and low activity (by the Spanish Short Version Minnesota leisure time Physical Activity Questionnaire). Detailed information can be found in previous publications [35,36]. The presence of at least three of five criteria was defined as frailty.

### Statistical Analysis

Statistical analyses were performed using IBM SPSS Statistics 26.0 (SPSS Inc) and JASP software 0.17.1 (JASP Team). Continuous variables have been presented as means and standard deviations if normally distributed or as medians and IQRs if not normally distributed. Frequencies and percentages were used for categorical data. The minimum estimated sample size was 200 participants per sex to detect an effect size equal to or higher than 0.80 area under the curve (AUC), with an α error of .05 and statistic power of 80% according to Bujang and Adnan [37].

#### Objective 1: Diagnostic Accuracy

The principle of parsimony was carried out to create multiple logistic regression equations with the smallest possible number of parameters, which allows adequate representation of the data. Therefore, multiple logistic regression equations stratified by sex with sarcopenia as the dependent variable and vertical power and calf circumference parameters as the independent variables were performed to create predictive equations to diagnose sarcopenia. The Hosmer-Lemeshow goodness-of-fit test was used to assess the calibration of the models. These analyses were presented for the 4 sarcopenia diagnostic criteria according to the EWGSOP2 guideline (Table 1). The models were internally validated using the bootstrap method by generating 1000 resampled data sets from the original cohort. Differences in performance (*c* statistic) between the initial and bootstrap models were checked using a paired sample design in order to correct for overoptimism if needed.

Then, receiver operating characteristic analyses were carried out using predictive probability from the results of the multiple logistic regression analyses to identify the best cutoff point for diagnosing sarcopenia. An AUC of 0.5 was interpreted as no discrimination, 0.7 to <0.8 as acceptable, 0.8 to 0.9 as excellent, and more than 0.9 as outstanding. These predictive equations were introduced into the *Sit to Stand* app with their respective cutoff points to automate diagnosis after video analysis.

Finally, participants were categorized as sarcopenic or healthy according to the index test (*Sit to Stand* app), and cross-tabulations with the reference standards were performed. Sensitivity, specificity, accuracy, positive and negative likelihood ratios, positive and negative predictive values, the number needed to get a false-positive result, and the number needed to get a false-negative result were calculated and expressed with their 95% CI. All statistical tests were 2-tailed.

#### Objective 2: Construct Validity

Associations between health-related outcomes (socioeconomic status, education level, smoking habit, polypharmacy, self-perceived health, comorbidities, depression, falls, and hospitalization) and the presence of frailty in those participants categorized as sarcopenic according to the index test (*Sit to Stand* app) were assessed using unadjusted and adjusted logistic regression analyses for age, sex, and BMI. The odds ratios (ORs) and their 95% CIs were calculated following a unidirectional rationale approach (1-tailed test).

## Results

### Participant Characteristics

A total of 775 older adults from 11 elderly social centers of the Region of Murcia (Spain) were screened for eligibility. Seventy-six participants reported at least one of the exclusion criteria (Mini-Cog test positive, n=59; implanted electronic devices, n=17). The data of 13 participants were lost due to unintentional video deletion (n=3), a lack of femur length measurement (n=5), and a lack of calf circumference measurement (n=5). Therefore, a total of 686 community-dwelling older adults aged between 60 and 88 years were finally included in the analysis (Table 2).

Sarcopenia prevalence ranged from 1.7% to 11.3% in women and 2.5% to 10.7% in men, depending on the diagnostic criteria proposed by the EWGSOP2 guideline.

**Table 2:** Characteristics of the participants (N=686).

Outcome			Women		Men
Participants, n (%)			406 (59.2)		280 (40.8)
Age (years), median (IQR)			71 (68-75)		72 (68-76)
Height (m), mean (SD)			1.55 (0.06)		1.68 (0.06)
Weight (kg), median (IQR)			66.0 (59.6-73.2)		79.3 (71.6-88.5)
BMI (kg/m^2^), median (IQR)			27.4 (24.4-30.1)		28.1 (25.8-31.2)
ASM^a^ (kg), median (IQR)			14.4 (13.1-15.7)		20.2 (18.7-22.3)
SMI^b^ (kg/m^2^), median (IQR)			5.9 (5.5-6.4)		7.2 (6.7-7.7)
**Calf circumference (m)^c^, median (IQR)**			0.36 (0.34-0.38)		0.37 (0.35-0.39)
	P25	0.34		0.35	
	P50	0.36		0.37	
	P75	0.38		0.39	
Femur length (m), median (IQR)			0.36 (0.34-0.38)		0.39 (0.36-0.4)
Handgrip strength (kg), mean (SD)			22.6 (4.3)		37.5 (6.8)
5STS^d^ (s), median (IQR)			12.1 (10.6-14.1)		12.2 (10.7-14.2)
**Vertical power (W/kg)^c^, mean (SD)**			5.0 (1.1)		5.89 (1.0)
	P25	4.5		5.4	
	P50	5.1		6.0	
	P75	5.7		6.6	
**Physical activity levels, n (%)**					
	Sedentary	22 (5.4)		42 (15.0)	
	Physically active	392 (93.8)		239 (85.1)	
**Sarcopenia prevalence, n (%)**					
	SARC_HG_^e^_+ASM_	20 (4.9)		11 (3.9)	
	SARC_HG+SMI_	7 (1.7)		7 (2.5)	
	SARC_5STS+ASM_	46 (11.3)		30 (10.7)	
	SARC_5STS+SMI_	18 (4.4)		24 (8.6)	
Frailty phenotype, n (%)			11 (2.7)		10 (3.6)

^a^ASM: appendicular skeletal mass.

^b^SMI: skeletal muscle index.

^c^Percentiles are shown as P25, P50, and P75.

^d^5STS: 5 times sit-to-stand.

^e^HG: handgrip strength.

### Objective 1: Diagnostic Accuracy

A total of 4 predicted equations per sex were created and introduced into the app with their respective cutoff points for sarcopenia diagnosis (Multimedia Appendix 1). The predicted models were well calibrated, and bootstrapping revealed stability of the models with differences in performance between the original and corrected *c* statistic lower than 0.004, resulting in the absence of overoptimism (Multimedia Appendix 2).

These predicted equations showed excellent ability (AUC range: 80.5%-87.1%) to discriminate between individuals with sarcopenia and apparently healthy individuals among both women and men. Sensitivity ranged from 0.70 to 0.83, whereas specificity ranged from 0.77 to 0.95, depending on the diagnostic criterion proposed by the EWGSOP2 guideline (Table 3). Sensitivity was higher when sarcopenia was defined according to the 5STS test instead of the handgrip strength test, whereas specificity was very similar between the diagnostic criteria used. Cross-tabulations of the index test and the reference standards of sarcopenia can be found in Multimedia Appendix 3.

**Table 3:** Diagnostic accuracy for detecting sarcopenia through the Sit to Stand app in women and men (N=686).

Gender and variable			Diagnostic criteria for sarcopenia						
			SARC_HG_^a^_+ASM_^b^		SARC_HG+SMI_^c^		SARC_5STS_^d^_+ASM_		SARC_5STS+SMI_
**Women (n=406)**									
	Cutoff point^e^	0.0503135		0.0378703		0.1223178		0.0638602	
	AUC^f^ (%), value (95% CI)	80.8 (71.3-90.3)		84.3 (69.7-98.9)		80.5 (73.2-87.9)		85.7 (76.4-95.0)	
	Sensitivity (%), value (95% CI)	70.0 (48.1-85.5)		71.4 (35.9-91.8)		71.7 (57.5-82.7)		77.8 (54.8-91.0)	
	Specificity (%), value (95% CI)	77.2 (72.8-81.1)		89.5 (86.1-92.1)		78.1 (73.5-82.0)		86.1 (82.3-89.2)	
	Accuracy (%), value (95% CI)	76.8 (72.4-80.9)		89.2 (85.7-92.0)		77.3 (73.0-81.3)		85.7 (81.9-89.0)	
	PLR^g^, value (95% CI)	3.1 (2.2-4.3)		6.8 (3.9-11.7)		3.3 (2.5-4.3)		5.6 (3.9-7.9)	
	NLR^h^, value (95% CI)	0.4 (0.2-0.8)		0.3 (0.1-1.0)		0.4 (0.2-0.6)		0.3 (0.1-0.6)	
	PPV^i^ (%), value (95% CI)	13.7 (10.2-18.3)		10.6 (6.4-17.1)		29.5 (24.2-35.3)		20.6 (15.4-26.9)	
	NPV^j^ (%), value (95% CI)	98.0 (96.2-99.0)		99.4 (98.2-99.8)		95.6 (93.1-97.1)		98.8 (97.2-99.5)	
	NNM_FP_^k^ (1:100), value (95% CI)	21.7 (17.7-25.7)		10.3 (7.4-13.3)		19.5 (15.6-23.3)		13.3 (10.0-16.6)	
	NNM_FN_^l^ (1:100), value (95% CI)	1.5 (0.3-2.7)		0.5 (0.0-1.2)		3.2 (1.5-4.9)		1.0 (0.0-1.9)	
**Men (n=280)**									
	Cutoff point^e^	0.0915922		0.0736029		0.1735792		0.1437517	
	AUC (%), value (95% CI)	84.8 (72.4-97.2)		85.1 (71.6-98.6)		87.1 (81.0-93.3)		86.8 (79.6-93.9)	
	Sensitivity (%), value (95% CI)	72.7 (43.4-90.3)		71.4 (35.9-91.8)		80.0 (62.7-90.5)		83.3 (64.2-93.3)	
	Specificity (%), value (95% CI)	94.4 (91.4-96.9)		94.9 (91.6-96.9)		87.6 (82.9-91.1)		86.7 (82.0-90.3)	
	Accuracy (%), value (95% CI)	93.6 (90.0-96.1)		94.3 (90.9-96.7)		86.8 (82.3-90.5)		86.4 (81.8-90.2)	
	PLR, value (95% CI)	13.0 (7.1-24.0)		13.9 (7.0-27.8)		6.4 (4.4-9.4)		6.3 (4.4-9.0)	
	NLR, value (95% CI)	0.3 (0.1-0.8)		0.3 (0.1-0.9)		0.2 (0.1-0.5)		0.2 (0.1-0.5)	
	PPV (%), value (95% CI)	34.8 (22.5-49.5)		26.3 (15.2-41.7)		43.6 (34.7-52.9)		37.0 (29.1-45.8)	
	NPV (%), value (95% CI)	98.8 (97.0-99.5)		99.2 (97.6-99.8)		97.4 (94.7-98.7)		98.2 (95.7-99.3)	
	NNM_FP_ (1:100), value (95% CI)	5.4 (2.7-8.0)		5.0 (2.4-7.6)		11.1 (7.4-14.7)		12.1 (8.3-16.0)	
	NNM_FN_ (1:100), value (95% CI)	1.1 (0.0-2.3)		0.7 (0.0-1.7)		2.1 (0.4-3.8)		1.4 (0.0-2.8)	

^a^HG: handgrip strength.

^b^ASM: appendicular skeletal mass.

^c^SMI: skeletal muscle index.

^d^5STS: 5 times sit-to-stand.

^e^Cutoff points are based on predictive probability as follows: 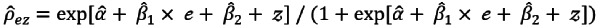, where 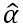, 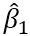, and 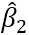 are the estimated regression coefficients (Multimedia Appendix 1), and *e* and *z* are the independent variables (ie, vertical power and calf circumference).

^f^AUC: area under the curve.

^g^PLR: positive likelihood ratio.

^h^NLR: negative likelihood ratio.

^i^PPV: positive predictive value.

^j^NPV: negative predictive value.

^k^NNM_FP_: number needed to get a false-positive result.

^l^NNM_FN_: number needed to get a false-negative result.

The accuracy of the index test was higher than 76% for women and higher than 86% for men. Among each 100 individuals tested, 10 to 22 women and 5 to 12 men could get a false-positive result, depending on the diagnostic criterion used by the EWGSOP2. The mean vertical power among those participants who got a false-positive result was lower than the 20th percentile of vertical power for women (<4.23 W/Kg) and the 15th percentile of vertical power for men (<4.92 W/Kg), whereas the mean calf circumference was lower than the 26th percentile for women (<34.2 cm) and the 15th percentile for men (<34.1 cm) in the sample analyzed.

Additionally, among each 100 individuals tested, 0 to 3 women and 1 to 2 men could get a false-negative result. The mean vertical power in those participants who got a false-negative result regardless of the diagnostic criterion used was 4.9 W/kg for women and 6.1 W/kg for men, whereas the mean calf circumference was >36.2 cm for women and >36.3 cm for men.

### Objective 2: Construct Validity

Overall, those individuals diagnosed with sarcopenia according to the index test (*Sit to Stand* app) showed more odds of having health-related adverse outcomes compared to their respective counterparts, regardless of the diagnostic criterion proposed by the EWGSOP2 guideline.

In our adjusted analysis, SARC_5STS+SMI_ was the diagnostic criterion most associated with health-related adverse outcomes. Individuals with sarcopenia according to the SARC_5STS+SMI_ criterion showed more odds of having a low education level (OR 1.69, 95% CI 1.15-2.48), a low socioeconomic level (OR 2.13, 95% CI 1.27-3.58), comorbidities (OR 3.74, 95% CI 2.15-6.50), a smoking habit (OR 1.83, 95% CI 1.03-3.27), polypharmacy (OR 2.80, 95% CI 1.80-4.34), and low self-perceived health (OR 2.32, 95% CI 1.48-3.62). Similar relationships were presented for SARC_5STS+ASM_ with the exception of current smoking habit. The SARC_HG+ASM_ criterion showed associations with a low education level (OR 1.48, 95% CI 1.03-2.14), comorbidities (OR 2.56, 95% CI 1.49-4.39), polypharmacy (OR 2.32, 95% CI 1.53-3.51), and low self-perceived health (OR 1.92, 95% CI 1.27-2.91). Likewise, similar relationships were presented for SARC_HG+SMI_ with the inclusion of two or more falls in the last year (OR 1.79, 95% CI 1.00-3.23).

All individuals diagnosed with sarcopenia according to the index test showed more odds of having frailty, regardless of the diagnostic criterion proposed by the EWGSOP2. In our adjusted analysis, SARC_HG+SMI_ was the diagnostic criterion most strongly associated with frailty (OR 6.64, 95% CI 2.61-16.87). Further details are provided in Table 4.

**Table 4:** Associations of sarcopenia determined by the index test (Sit to Stand app) with health-related risk factors and the frailty phenotype (N=686).

Risk factor			Diagnostic criteria for sarcopenia																	
			SARC_HG_^a^_+ASM_^b^					SARC_HG+SMI_^c^					SARC_5STS_^d^_+ASM_					SARC_5STS+SMI_		
			OR^e^ (95% CI)		Adjusted OR (95% CI)		OR (95% CI)			Adjusted OR (95% CI)		OR (95% CI)			Adjusted OR (95% CI)		OR (95% CI)			Adjusted OR (95% CI)
**Education level**																				
	High level (n=356; reference)																			
	Low level (n=319)	1.71 (1.23-2.39)^f^		1.48 (1.03-2.14)^f^		2.19 (1.40-3.43)^f^			1.93 (1.18-3.14)^f^		1.76 (1.31-2.38)^f^			1.68 (1.21-2.35)^f^		1.62 (1.15-2.27)^f^			1.69 (1.15-2.48)^f^	
**Socioeconomic level**																				
	High level (n=545; reference)																			
	Low level (n=97)	1.55 (1.01-2.40)^f^		1.21 (0.75-1.96)		1.75 (0.10-2.99)^f^			1.40 (0.77-2.56)		1.95 (1.32-2.88)^f^			1.89 (1.22-2.94)^f^		1.85 (1.21-2.84)^f^			2.13 (1.27-3.58)^f^	
**Comorbidities**																				
	<2 pathologies (n=159; reference)																			
	≥2 pathologies (n=527)	3.03 (1.82-5.03)^f^		2.56 (1.49-4.39)^f^		5.17 (2.18-12.24)^f^			5.03 (2.05-12.30)^f^		2.87 (1.87-4.43)^f^			2.94 (1.85-4.67)^f^		2.93 (1.76-4.87)^f^			3.74 (2.15-6.50)^f^	
**Smoking habit**																				
	Not smoking (n=611; reference)																			
	Smoking (n=74)	0.67 (0.38-1.21)		1.28 (0.67-2.41)		0.65 (0.29-1.44)			1.10 (0.46-2.63)		0.99 (0.62-1.60)			1.60 (0.94-2.70)		1.31 (0.80-2.16)			1.83 (1.03-3.27)^f^	
**Polypharmacy**																				
	<5 drugs (n=538; reference)																			
	≥5 drugs (n=145)	2.03 (1.41-2.92)^f^		2.32 (1.53-3.51)^f^		2.52 (1.61-3.96)^f^			2.92 (1.74-4.91)^f^		2.23 (1.60-3.11)^f^			2.62 (1.79-3.84)^f^		2.12 (1.47-3.05)^f^			2.80 (1.80-4.34)^f^	
**Self-perceived health**																				
	High level (n=553; reference)																			
	Low level (n=133)	2.14 (1.48-3.10)^f^		1.92 (1.27-2.91)^f^		2.29 (1.44-3.64)^f^			2.29 (1.37-3.85)^f^		1.89 (1.34-2.66)^f^			1.96 (1.33-2.88)^f^		1.81 (1.24-2.65)^f^			2.32 (1.48-3.62)^f^	
**Depression**																				
	Not depressed (n=557; reference)																			
	Depressed (n=129)	1.74 (1.19-2.54)^f^		1.42 (0.93-2.15)		1.57 (0.96-2.58)			1.36 (0.79-2.33)		1.52 (1.07-2.17)^f^			1.39 (0.94-2.04)		1.29 (0.86-1.92)			1.29 (0.81-2.03)	
**Falls in the last year**																				
	<2 falls (n=599; reference)																			
	≥2 falls (n=85)	1.68 (1.08-2.62)^f^		1.31 (0.73-2.33)		2.07 (1.21-3.55)^f^			1.79 (1.00-3.23)^f^		1.63 (1.08-2.45)^f^			1.46 (0.93-2.30)		1.39 (0.87-2.20)			1.37 (0.80-2.32)	
**Hospitalization**																				
	Not hospitalized (n=594; reference)																			
	Hospitalized (n=90)	1.23 (0.78-1.95)		1.27 (0.80-2.12)		1.36 (0.76-2.43)			1.15 (0.61-2.17)		1.57 (1.05-2.35)^f^			1.50 (0.95-2.35)		1.84 (1.19-2.83)^f^			1.49 (0.90-2.48)	
**Frailty phenotype**																				
	Not frail (n=665; reference)																			
	Frail (n=21)	4.35 (2.08-9.10)^f^		3.95 (1.69-9.27)^f^		6.44 (2.97-13.95)^f^			6.64 (2.61-16.87)^f^		3.59 (1.72-7.48)^f^			3.32 (1.44-7.67)^f^		2.97 (1.39-6.35)^f^			3.42 (1.38-8.59)^f^	

^a^HG: handgrip strength.

^b^ASM: appendicular skeletal mass.

^c^SMI: skeletal muscle index.

^d^5STS: 5 times sit-to-stand.

^e^OR: odds ratio.

^f^Statistical significance at an α level of .05 (*P*<.05; 1-tailed test).

## Discussion

### Principal Findings

This diagnostic accuracy study involving 686 community-dwelling older adults from elderly social centers revealed that sit-to-stand kinematic analysis through a video analysis–based app together with a simple measure of calf circumference had excellent ability (AUC range: 80.5%-87.1%) to discriminate between individuals with sarcopenia and apparently healthy individuals, regardless of the diagnostic criterion proposed by the EWGSOP2 guideline. Additionally, people categorized as sarcopenic through the *Sit to Stand* app showed more odds of having health-related adverse outcomes and frailty compared to their respective counterparts.

Kinematic variables derived from the sit-to-stand test, such as vertical velocity and vertical power, have been widely associated with several measures of physical function in older adults [20,21,25,38]. These variables have also demonstrated their potential to discriminate between apparently healthy older adults and those with a history of falls, sarcopenia, or even frailty [16-19]. However, these analyses have been usually performed through specialized equipment in laboratory settings, which are not affordable, require technical expertise, and are time-consuming to analyze, limiting their potential to be used in clinical settings. Our results derived from the *Sit to Stand* app showed a similar ability to discriminate between individuals with sarcopenia and apparently healthy older adults compared with the ability reported in previous studies using force plates (AUC range: 85.8%-90.6%) [17], but with the advantage of an easy-to-use interface and automatic data processing from the app, which allows health care professionals to screen for sarcopenia in short time periods (<5 min), emphasizing its clinical utility.

Previous validated questionnaires for identifying sarcopenia cases in short time periods, such as the SARC-F or its modified version adding calf circumference, have been recommended for identifying sarcopenia cases by the international sarcopenia guideline [39] and 2 expert consensus guidelines (the EWGSOP2 and the Asian Working Group for Sarcopenia-2019) [1,40], owing to their affordability, rapid and simple assessment, and association with adverse clinical outcomes, which represent advantages to be used in clinical settings. However, a recent systematic review and meta-analysis provided strong evidence that their use in clinical practice could be nonoptimal mainly due to their low to moderate sensitivity (29%-57%) despite moderate to high specificity (69%-91%), independent of the sarcopenia definition [27]. This issue would lead to an underdiagnosis of individuals with sarcopenia, predisposing them to progress toward adverse health-related consequences [27]. The sensitivity of the *Sit to Stand* app ranged from 70% to 83.3%, with a low false-negative rate and high negative predictive values ranging from 95.6% to 99.4%, depending on the diagnostic criteria used. In other words, among each 100 individuals tested, 0 to 3 women and 1 to 2 men could get a false-negative result according to the different diagnostic criteria by the EWGSOP2 guideline. These results proved the exclusion of sarcopenia in cases of a negative test result, showing that the app was quite reliable to rule out the disease.

Recent studies have reported differences in prevalence between the diagnostic criteria proposed by the EWGSOP2 guideline, showing a more than 2-fold prevalence when the 5STS test result was used as a low muscle strength criterion instead of handgrip strength in European community-dwelling older adults [6,41]. In our study, sarcopenia prevalence ranged from 2% to 11%, with a higher value when the 5STS test result was used as a diagnostic criterion compared to handgrip strength. The low prevalence rate found in our study could be due to the characteristics of the sample analyzed, with participants being recruited from elderly social centers where movement-based activities are encouraged. In fact, only 10% of the participants were sedentary, while most of them were moderately active. It is well known that physical activity has a protective effect against sarcopenia, reducing the odds of its development by up to 55% [42]. The development of a valid diagnostic test with enough sensitivity in low prevalence settings is a challenge. If the setting includes individuals with sarcopenia having a more advanced disease, there may be fewer false-negative results than in settings where individuals are unsuspected of having the disease, and sensitivity may be overestimated [43]. Despite the low prevalence rate, the *Sit to Stand* app showed moderate to high sensitivity, with a better value when the 5STS test result was used as a diagnostic criterion instead of handgrip strength possibly due to the higher prevalence found.

The app was validated against the different criteria proposed by the EWGSOP2 guideline since it has been recently reported that there is scarce overlap in people with sarcopenia when different diagnostic criteria are used [6,41,44], that is, some people diagnosed as having sarcopenia using the handgrip strength criterion were categorized as healthy using the 5STS criterion or vice versa, leading to underdiagnosis of up to 10% [6]. Additionally, recent studies have reported that the relationships between sarcopenia and adverse clinical outcomes could be dependent on the diagnostic criteria used [6,45,46]. Chew et al [46] showed that the 5STS test result had the best predictive validity for adverse outcomes at 2 years compared to handgrip strength in community-dwelling older adults with sarcopenia. Our results indicated that those individuals diagnosed with sarcopenia according to the index test (*Sit to Stand* app) showed more odds of having health-related adverse outcomes. These relationships were very consistent between the diagnostic criteria proposed by the EWGSOP2 guideline. Moreover, all individuals diagnosed with sarcopenia according to the index test showed more odds of having frailty, regardless of the diagnostic criterion used. It was not surprising since transitions from robustness to frailty status according to the Fried frailty phenotype are more frequent in community-dwelling older adults with sarcopenia than in those without sarcopenia [10]. These results highlight the validity of our index test for predicting adverse outcomes. This app could serve as a simple clinical tool for sarcopenia screening to potentially prevent the risk of further adverse events and the progression to severe sarcopenia, as well as the transition to other conditions such as frailty. This is of great importance since the combination of sarcopenia and frailty could lead to a higher risk of developing disability [47] and mortality [48] than either condition alone.

In our index test, false-positive results were mainly associated with a reduced muscle power below the 20th percentile or a reduced calf circumference below the 26th percentile (<34.2 cm) in the sample analyzed. A reduction in muscle power below the 33rd percentile has been previously shown to be associated with disability in activities of daily living and reduced physical performance to a greater extent than probable or confirmed sarcopenia [49]. Moreover, muscle power below the 33rd percentile has been shown to be independently associated with mortality and hospitalization in older adults [50]. Likewise, reduced calf circumference has been previously shown to be related to a higher frailty index and lower functional performance and strength [51]. Furthermore, calf circumference below 34.5 cm has been reported to be independently associated with an increased risk of mortality during 9 years of follow-up in community-dwelling older adults [52]. Therefore, our false-positive individuals probably had a higher risk of developing health-related adverse outcomes than their respective counterparts. Thus, despite the moderate rate of false-positive results from the *Sit to Stand* app, these individuals should not go unnoticed with the aim of treatment through appropriate interventions, such as resistance training and optimal dietary intake, to improve muscle strength, muscle power, and muscle quantity as recommended [53]. The fact that the criteria for sarcopenia diagnosis in our index test were based on reduced muscle power and calf circumference, which have been widely associated with health adverse consequences, emphasizes the use of this app in clinical settings where muscle strength dynamometers or muscle mass measurement techniques are usually limited or restricted [12]. Furthermore, since muscle power declines with aging at an earlier and faster rate compared with muscle mass and strength [23,24], which are the base factors of the definition of sarcopenia, this app could potentially detect the early stages of sarcopenia where declines in muscle strength or mass are still not recognized. Thus, detecting reduced muscle power below a specific threshold might be a sign to start an appropriate intervention in these individuals. Further studies are needed to independently evaluate the validity of this app in community-dwelling older adults and to define a specific threshold of reduced vertical power using this app and its associated risk with health-related adverse outcomes.

### Limitations

One of the main limitations of our study is the low prevalence rate of individuals with sarcopenia at elderly social centers. This situation led to imprecise estimates of the sensitivity parameter as can be observed in its wide CIs. This issue is usually reported in diagnostic studies with a low prevalence rate [43]. However, there is a need to develop diagnostic tools in those settings where sarcopenia could be overlooked or is uncommon as anticipatory strategies for detecting the disease and preventing health adverse consequences in community-dwelling older adults. Our predicted models were well calibrated, and bootstrapping revealed stability of the models, but further studies are needed to confirm the validity of our results. The app also showed a relationship with health-related risk factors, but these outcomes were self-reported in a cross-sectional time point. Therefore, the information collected depended on the subject’s perception, and cross-sectional data do not allow causal relationships between measures. Finally, muscle quantity was assessed by bioelectrical impedance analysis, which is not considered as the gold standard for the assessment of this variable. In order to overcome this limitation, a cross-validated equation in a sample with similar characteristics was used as previously recommended [1,34].

### Conclusions

The *Sit to Stand* app appears to have good diagnostic performance for detecting sarcopenia in well-functioning Spanish community-dwelling older adults. Individuals with sarcopenia diagnosed by the video analysis–based app showed more odds of having health-related risk factors and frailty compared to their respective counterparts, regardless of the diagnostic criteria proposed by the EWGSOP2 guideline. Further studies are needed to independently evaluate the validity of this app in community-dwelling older adults.

## Appendix

Multimedia Appendix 1 Multiple logistic regression equations stratified by sex.

Multimedia Appendix 2 Differences in performance (c statistic) between the bootstrap models and the initial models stratified by sex.

Multimedia Appendix 3 Cross-tabulation of the index test (Sit to Stand app) and the reference standards of sarcopenia according to the European Working Group on Sarcopenia in Older People-2 (EWGSOP2) guideline in women and men (N=686).

## Abbreviations

5STS: 5 times sit-to-stand
ASM: appendicular skeletal mass
AUC: area under the curve
EWGSOP2: European Working Group on Sarcopenia in Older People-2
OR: odds ratio
