# Supplementary material for: Sit-to-Stand Video Analysis–Based App for Diagnosing Sarcopenia and Its Relationship With Health-Related Risk Factors and Frailty in Community-Dwelling Older Adults: Diagnostic Accuracy Study
Source: J Med Internet Res. 2023 Dec 8;25:e47873. doi: 10.2196/47873 (PMC10746979; doi:10.2196/47873)
Supplement: Multimedia Appendix 1 [file jmir_v25i1e47873_app1.docx]

**Multimedia Appendix 1.** Multiple logistic regression equations stratified by sex.

|  |  | Men | | | |  | Women | | | |
| --- | --- | --- | --- | --- | --- | --- | --- | --- | --- | --- |
| Dependent variable | Independent variable | β | SE | Exp (β) | 95% CI |  | β | SE | Exp (β) | 95% CI |
| SARC_HG+ASM_ | Constant | 12.972 | 4.674 | 411433.3 |  |  | 9.711 | 3.14 | 16496.6 |  |
|  | Vertical power | –0.805 | 0.24 | 0.45 | 0.3 to 0.7 |  | –0.856 | 0.19 | 0.42 | 0.3 to 0.6 |
|  | Calf-circumference | –0.328 | 0.13 | 0.72 | 0.6 to 0.9 |  | –0.248 | 0.08 | 0.78 | 0.7 to 0.9 |
| SARC_HG+SMI_ | Constant | 11.537 | 5.26 | 102387.5 |  |  | 14.887 | 5.64 | 29188640.5 |  |
|  | Vertical power | –0.682 | 0.28 | 0.51 | 0.3 to 0.9 |  | –0.748 | 0.33 | 0.47 | 0.2 to 0.9 |
|  | Calf-circumference | –0.321 | 0.15 | 0.73 | 0.5 to 0.9 |  | –0.449 | 0.16 | 0.64 | 0.5 to 0.9 |
| SARC_5STS+ASM_ | Constant | 19.645 | 3.9 | 340224513.4 |  |  | 12.202 | 2.37 | 199241.3 |  |
|  | Vertical power | –0.921 | 0.2 | 0.4 | 0.3 to 0.6 |  | –0.841 | 0.15 | 0.43 | 0.3 to 0.5 |
|  | Calf-circumference | –0.464 | 0.1 | 0.63 | 0.5 to 0.8 |  | –0.291 | 0.06 | 0.75 | 0.6 to 0.8 |
| SARC_5STS+SMI_ | Constant | 19.02 | 4.1 | 182084090.9 |  |  | 17.516 | 3.93 | 40469541.1 |  |
|  | Vertical power | –0.749 | 0.2 | 0.47 | 0.3 to 0.7 |  | –0.569 | 0.22 | 0.57 | 0.4 to 0.9 |
|  | Calf-circumference | –0.482 | 0.1 | 0.62 | 0.5 to 0.8 |  | –0.519 | 0.11 | 0.59 | 0.5 to 0.7 |

HG: Handgrip strength; 5STS: Five-chair stand test; ASM: Appendicular Skeletal Mass; SMI: Skeletal Muscle Index; 95% CI: 95% Confidence Interval for β–exponential.
