# Supplementary material for: Sit-to-Stand Video Analysis–Based App for Diagnosing Sarcopenia and Its Relationship With Health-Related Risk Factors and Frailty in Community-Dwelling Older Adults: Diagnostic Accuracy Study
Source: J Med Internet Res. 2023 Dec 8;25:e47873. doi: 10.2196/47873 (PMC10746979; doi:10.2196/47873)
Supplement: Multimedia Appendix 2 [file jmir_v25i1e47873_app2.docx]

**Multimedia Appendix 2.** Differences in performance (*c* statistic) between the bootstrap models and the initial models stratified by sex.

| **Women** | | | | |
| --- | --- | --- | --- | --- |
| Diagnostic criterion | Initial model *c*-statistic | Bootstrap model *c-*statistic | *c*-statistic diff. | *P-*value |
| SARC_HG+ASM_ | 0.808 | 0.808 | 0.000 | 1 |
| SARC_HG+SMI_ | 0.843 | 0.843 | 0.000 | 1 |
| SARC_5STS+ASM_ | 0.805 | 0.806 | –0.001 | 0.235 |
| SARC_5STS+SMI_ | 0.857 | 0.856 | 0.001 | 0.217 |
| **Men** | | | | |
| Diagnostic criterion | Initial model *c*-statistic | Bootstrap model *c-*statistic | *c*-statistic diff. | *P-*value |
| SARC_HG+ASM_ | 0.848 | 0.848 | <0.001 | 0.617 |
| SARC_HG+SMI_ | 0.851 | 0.848 | 0.003 | 0.282 |
| SARC_5STS+ASM_ | 0.871 | 0.872 | <0.001 | 0.480 |
| SARC_5STS+SMI_ | 0.868 | 0.868 | 0.000 | 1 |

HG: Handgrip strength; 5STS: Five-chair stand test; ASM: Appendicular Skeletal Mass; SMI: Skeletal Muscle Index; *c*-statistic diff: Differences in *c-*statistic.
