# Supplementary material for: Sit-to-Stand Video Analysis–Based App for Diagnosing Sarcopenia and Its Relationship With Health-Related Risk Factors and Frailty in Community-Dwelling Older Adults: Diagnostic Accuracy Study
Source: J Med Internet Res. 2023 Dec 8;25:e47873. doi: 10.2196/47873 (PMC10746979; doi:10.2196/47873)
Supplement: Multimedia Appendix 3 [file jmir_v25i1e47873_app3.docx]

**Multimedia Appendix 3.** Cross-tabulation of the index test (*Sit to Stand* app) and the reference standards of sarcopenia according to the European Working Group on Sarcopenia in Older People-2 (EWGSOP2) guideline in women and men (N=686).

|  | **Women (n= 406)** | | | | | | | | | | |
| --- | --- | --- | --- | --- | --- | --- | --- | --- | --- | --- | --- |
|  | **SARC _HG+ASM_** | |  | **SARC _HG+SMI_** | |  | **SARC _5STS+ASM_** | |  | **SARC _5STS+SMI_** | |
| **Index test** | Presence | Absence |  | Presence | Absence |  | Presence | Absence |  | Presence | Absence |
| Presence | 14 | 88 |  | 5 | 42 |  | 33 | 79 |  | 14 | 54 |
| Absence | 6 | 298 |  | 2 | 357 |  | 13 | 281 |  | 4 | 334 |
|  | **Men (n= 280)** | | | | | | | | | | |
|  | **SARC _HG+ASM_** | |  | **SARC _HG+SMI_** | |  | **SARC _5STS+ASM_** | |  | **SARC _5STS+SMI_** | |
| **Index test** | Presence | Absence |  | Presence | Absence |  | Presence | Absence |  | Presence | Absence |
| Presence | 8 | 15 |  | 5 | 14 |  | 24 | 31 |  | 20 | 34 |
| Absence | 3 | 254 |  | 2 | 259 |  | 6 | 219 |  | 4 | 222 |

HG: Handgrip strength; 5STS: Five-chair stand test; ASM: Appendicular Skeletal Mass; SMI: Skeletal Muscle Index.
